# Identifying Patterns of Failure and Risk Factors for Recurrence in Patients of Paratesticular Sarcomas: Protocol of a Systematic Review and Meta-Analysis

**DOI:** 10.29337/ijsp.145

**Published:** 2021-05-28

**Authors:** Anil Gupta, Rashi Kulshrestha, Kamal Kishore, Oliver Micke, Rishabh Kumar, Kanika Garg, Dayanand Sharma, Rambha Pandey

**Affiliations:** 1 Department of Radiotherapy, All India Institute of Medical Sciences, New Delhi, IN; 2 Clinical Radiation Oncology fellowship program, Department of Radiation Oncology, BC Cancer Agency, Abbotsford, BC, CA; 3 Department of Biostatistics, Post Graduate Institute of Medical Education and Research, Chandigarh, IN; 4 Prof. Dr. med, Klinik für Strahlentherapie und Radioonkologie, Franziskus Hospital, Kiskerstrasse, Bielefeld, DE; 5 Department of Radiation Oncology, All India Institute of Medical Sciences, New Delhi, IN; 6 Department of Anaesthesiology, Employee’s State Insurance Corporation, Noida, IN; 7 Department of Radiotherapy, All India Institute of Medical Sciences, New Delhi & National Cancer Institute, Jhajjar, Haryana, IN; 8 Department of Radiotherapy, All India Institute of Medical Sciences, New Delhi, IN

**Keywords:** Protocol, systematic review, meta-analysis, paratesticular sarcoma, spermatic cord, epididymis

## Abstract

**Introduction:**

Para testicular sarcomas are rare mesenchymal tumors that affect patients of all ages. Unlike other sites of sarcoma, they tend to be of lower grade and have a higher propensity for lymphatic spread. Management is hampered by the small number of patients who differ in terms of tumor grade and histology. Current treatment approaches are based on case reports, small case series and literature reviews, resulting in a number of unresolved issues. The consensus on the type of surgery and adjuvant treatment is yet to be determined. The local relapse rates in the scrotum and groin after orchidectomy comes out to be 25%–37%, indicating the need for either aggressive surgery or adjuvant treatment. There is a paucity of data identifying the patterns of failure and risk factors for recurrence, which will help clinicians tailor appropriate treatment.

**Methods:**

We aim to perform a systematic review and meta-analysis of the available data in the last 50 years in a methodologically rigorous and transparent manner to identify patterns of failure and high-risk factors for recurrence. The protocol is prepared in accordance with the Preferred Reporting Items for Systematic Reviews and Meta-analyses (PRISMA-P) 2015 guidelines. The protocol is registered in the International Prospective Register of Systematic Reviews (CRD42021237134).

**Highlights:**

Para testicular sarcomas are rare mesenchymal tumors that affects patients of all ages. Current treatment approaches are based on case reports, small case series and literature reviews, resulting in a number of unresolved issues. A systemic review was performed in 2013 based on survival rates, prognostic factors, and relapse sites on paratesticular sarcomas. However, it lacks a comprehensive review that can guide radiation oncologists to select in which patient’s postoperative radiotherapy is warranted and define the target volume based on histopathological type, stage, and grade of the tumor. After 2013, new case series with improved methodology and sample size are published, which adds new information to the literature. In one case series, 22 patients with spermatic cord sarcoma were discussed, while in another study, long-term outcome analysis of 51 patients was discussed, and another study discussed eight patients.

## Introduction

Paratestis is an anatomical region surrounding the testis, and it includes the spermatic cord and its contents, vas deferens, testicular tunics, epididymis, and efferent ductules [1]. The majority of paratesticular abnormalities are benign, including lipoma, hydrocele, leiomyomas, and inflammatory conditions such as epididymitis. Various cystic and proliferative lesions are also observed. Soft tissue sarcomas account for approximately 1% of all malignant tumors [2], among these paratestis is an unusual sight, which accounts for 1% of soft tissue sarcomas [3], and is still the most common malignancy in this anatomical region. These tumors may arise from epididymis, spermatic cord, or the mesenchymal sheaths surrounding the testicle, with spermatic cord being the major site (75–90% of the cases) [4].

Paratesticular masses typically present as painless scrotal swelling, with or without a hydrocele. Differentiating benign lesions from malignant lesions is often difficult. Malignant swelling is often accompanied by rapid growth, symptomatic presentation and poorly defined solid masses with heterogeneity. While benign inguinoscrotal swellings, such as hernias and hydroceles, can be diagnosed on clinical examination, all atypical swellings should be thoroughly investigated before definitive management/surgical exploration/surgical resection.

Ultrasound is the first investigation of choice for the detection and evaluation of paratesticular masses; it has a high sensitivity to reliably differentiate between intratesticular and extratesticular lesions and is easily available and inexpensive. Computed tomography (CT) and magnetic resonance (MR) imaging help distinguish a paratesticular mass from a retroperitoneal lesion extending into the scrotum. CT allows us to define the morphology and helps in staging, while the multiplanar imaging capability of MR can achieve a more precise localisation, including its anatomic relationship to the surrounding structures. The diagnostic role of CT and MR imaging influences the surgical management as well, in malignant lesions.

Based on the characteristics of mature mesenchymal tissues, distinct biological characteristics and behaviors there are more than 50 different histological types of para testicular soft tissue sarcomas. The most common tumor types in adults are well-differentiated liposarcomas and leiomyosarcomas (LMS), while malignant fibrous histiocytoma (MFH) and fibrosarcoma are rare [2]. Leiomyosarcoma is the most frequent histological subtype in older patients and has been postulated to arise from the muscular components of the paratestis. In addition, the prognosis and treatment of most sarcomas are closely related to their anatomical location.

Simple excision is considered inadequate for paratesticular sarcomas, as re-excision surgeries with wide margins have revealed microscopic residual disease in approximately 27% of completely excised cases [5]. Sarcomas have a propensity to infiltrate local tissues widely, making complete resection difficult. A decrease in local recurrence has been observed in patients who underwent wide local excision after a prior incomplete resection [6]. Therefore, aggressive surgical strategies are recommended for the management of paratesticular sarcomas Hemiscrotectomy is advised in cases where scrotal skin is involved or a previous surgical scar is present. Recurrences are found to be higher in patients who underwent unplanned “exploration” procedures with unexpected malignant sarcoma pathologies, as opposed to those who underwent elective resection procedures.

The usefulness of adjuvant treatment remains controversial. Despite complete resection, these tumours have a higher recurrence rate (21%) [7]. Radical orchiectomy alone may be an inadequate therapy [8,9].

The role of retroperitoneal lymph node dissection (RPLND) in paratesticular sarcomas remains debatable. Nodal failure in the retroperitoneum has been reported in 14%–29% of paratesticular tumors [10]. On the contrary, a report from the MD Anderson suggests that the risk of nodal failure is low, similar to most extremity soft-tissue sarcoma histology [11]. If the rates of nodal failure are low, the extent of the pelvic radiation portals can be minimised and the reduced margins of the surgical resection can decrease the potential treatment toxicity. According to Hazariwala et al “such a study would require a large patient pool to adequately power the endpoints adequately and, given the rarity of these sarcomas, it is unlikely that such a study will be conducted” [12]. This rarity can be seen in a multicentric prospective study conducted in Italy, where only 9 cases of paratesticular sarcoma were available over a long span of 18 years [13].

Recognising patterns of failure of already treated cases may help in understanding the shortcomings of previous treatments and may guide in tailoring a more robust treatment. Various case series have mentioned patterns of failure [5,6] while others have shown the benefits of adjuvant treatment [12,14,15].

A systemic review was performed in 2013 based on survival rates, prognostic factors, and relapse sites on paratesticular sarcomas [16]. However, it lacks a comprehensive review that can guide radiation oncologists to select patients for whom postoperative radiotherapy is warranted and define the target volume based on histopathological type, stage, and grade of the tumor. After 2013, new case series with improved methodology and sample size are published, which adds new information to the literature. In one case series, 22 patients with spermatic cord sarcoma were discussed [17], while in another study, long-term outcome analysis of 51 patients was discussed [18], and another study discussed eight patients [19].

In the absence of a contemporary internally valid study and with the availability of new evidence, we aim to systematically review the literature in a methodologically rigorous and transparent manner.

The protocol is prepared in accordance with the Preferred Reporting Items for Systematic Reviews and Meta-analyses (PRISMA-P) 2015 guidelines [20]. These guidelines are created by an international group of experts to improve the transparency, accuracy, completeness, and frequency of documented systematic reviews and meta-analysis protocols. The protocol is registered in the International Prospective Register of Systematic Reviews (PROSPERO) [CRD42021237134]. The systematic review will follow guidelines of the Cochrane as mentioned in Cochrane Handbook for Systematic Reviews of Interventions [21].

## Objectives

This systematic review aims to evaluate the patterns of failure and risk factors for the recurrence of paratesticular sarcoma after surgical excision and adjuvant treatment. To this end, the proposed systematic review and meta-analysis will answer the following questions:

1. The patterns of failure post-treatment were mainly classified into three categories: local recurrence (LR), regional and distant metastasis (DM). LR was defined as the first recurrence of the disease histology type at the primary tumor site. Regional recurrence is the recurrence of inguinal and/or retroperitoneal lymph nodes. DM was defined as a recurrent disease at a distant site or multiple intra-abdominal recurrences.
2. To be able to identify high risk factors for recurrences post treatment, which may help clinicians in identifying poor prognostic factors.
3. To assess the effect of adjuvant treatment such as radiotherapy/and or chemotherapy, and whether, adjuvant treatment was able to reduce recurrences or not.
4. To present descriptive analysis, which will depict patient characteristics and tumor characteristics.

### Materials and methods

#### Study designs

All available studies, such as case reports, case series, case-control studies, and randomised control trials, will be included in this study.

##### Population (P)

Adult patients (≥18 years) diagnosed with paratesticular sarcoma, including the contents of the spermatic cord, testicular tunics, epididymis and vestigial remnants, such as the appendices epididymis and testis. We will exclude studies involving exclusively non-adult patients (<18 years), older patients (age >75 years), systemic disease (M1), non-sarcoma histology (predominant), second malignancy, synchronous malignancy and recurrence from other sites.

##### Intervention (I)

Surgical excision without neo/and or adjuvant therapy.

##### Comparison (C)

Surgical treatment with neo/and or adjuvant therapy.

##### Outcomes and prioritization (O)

The primary outcome will be recurrence-free survival (RFS), local control (LC), failure patterns after treatment, and high-risk factors for recurrence. Patient parameters (such as age), treatment parameters (such as type of surgery, surgical resection margins, and adjuvant treatment), and disease parameters (such as size, histology, and grade) influence treatment. We have multiple parameters to inspect and identify its role in the failure of treatment failure. Failure refers to disease recurrence and can occur locally, regionally or distantly. Identifying the influence of variables on outcomes will help tailor treatment strategies and reduce recurrences.

The secondary outcome will be to calculate mean age, geographical distribution, mean tumor size, most common histology, most common grade, survival outcomes such as disease-specific survival (DSS) and overall survival (OS).

##### Time (T)

Studies published from the 1970 onwards till end of 2020 meeting the inclusion criteria will be taken for review. Studies will be selected for inclusion based on the length of follow-up of the outcomes. The following will be used as a guide for all the study designs.

1. For all decision-making endpoint outcomes, studies should have a follow-up time of at least 6 month.
2. For all surrogate outcomes, studies should be at least 6 months duration for follow-up

##### Setting

There will be no restrictions by the type of setting.

##### Language

Due to the rarity of the disease and limited literature, we will include articles reported in all languages. Google translator, a web-based tool for language translation, will be used to translate all articles into English.

**Figure 1:**
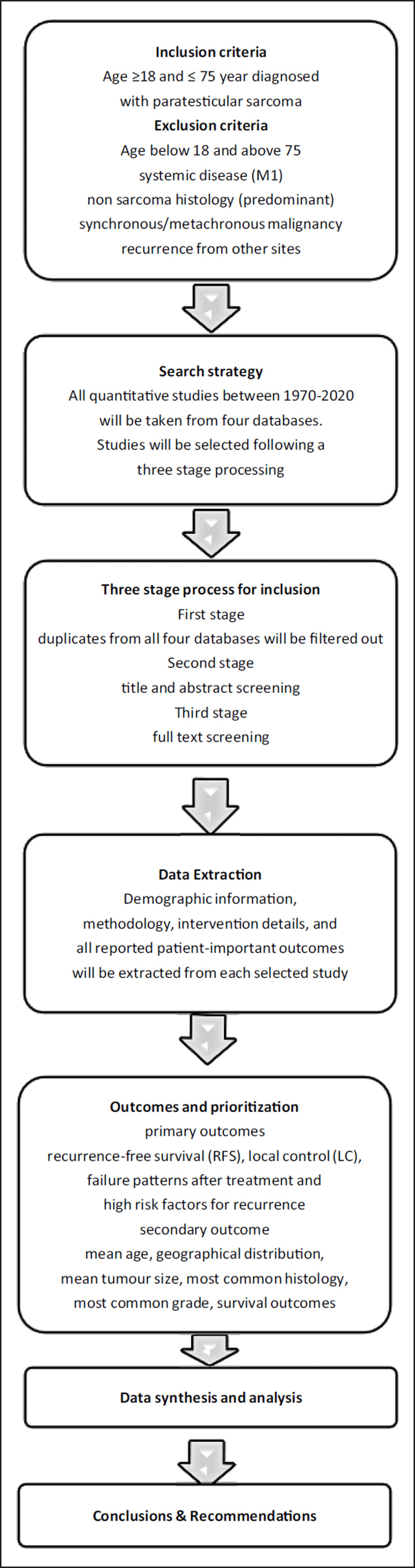
Conceptual Framework.

To sum up, patients with non-metastatic paratesticular sarcoma (P), the failure rate (O) with surgical excision (I) will be compared to adjuvant therapy (C) in last 5 decades (T). ***Figure 1*** depicts the conceptual framework of the study.

## Search Strategy

All quantitative studies will be sought. No study design and language limits will be imposed on the search, although only studies in languages other than English that can be translated adequately using Google Translate will be included due to limited resources. A time filter for the year 1^st^ January 1970 until the current date of extraction of the literature 2020 will be imposed. The peer review of electronic search strategies (PRESS) 2015 guidelines [22] will be used to enhance the quality and comprehensiveness of the electronic literature search. Studies will be taken from 4 databases 1) Pubmed 2) Web of Science 3) Embase 4) Scopus. The International Clinical Trials Registry Platform Search Portal and ClinicalTrials.gov will be searched for ongoing or recently completed trials. PROSPERO will be searched for ongoing or recently completed systematic reviews. As relevant studies are identified, reviewers will check for additional relevant cited and citing articles.

Keywords and filter used are given in supplementary file 1.

## Study records

The literature search results will be uploaded to Covidence (covidence.org). It is a web-based software platform which streamlines the production of systematic reviews. Studies will be selected for inclusion following a three-stage process using Covidence.

In the first stage, duplicates from all four databases will be filtered out. In the second stage, two independent reviewers will screen the title and abstract of all the studies. Studies not meeting the eligibility criteria will be excluded. Conflict or discrepancy will be resolved through mutual discussion. In the third stage of screening, full-text manuscripts of all screened studies from the second stage will be retrieved. The final inclusion or exclusion decisions will be made by examining of the full-text manuscripts. Two reviewers will then independently select studies that meet the predefined criteria. All disagreements will be discussed and resolved by an expert review author. The reason for exclusion will be recorded. A flow chart of included and excluded studies at various stages of selection will be made following the PRISMA 2009 flow diagram (supplementary file 2). In multi-country studies, the data will be extracted for each country. However, if data are not reported country-wise, the authors will be contacted to request country-wise data.

## Data extraction

A standardised data extraction form (supplementary file 3) is designed which will be used for the data extraction. It will be uploaded in Covidence software and two authors (AG and RK) will independently perform data screening and extraction. Any conflicts arising during the data extraction process will be resolved by discussion and consensus involving the two authors or if deemed necessary a senior review author (DNS, OM or RP) will arbitrate. Information will be extracted regarding demographic information, methodology, intervention details, and all important reported patient outcomes. Individual studies may consist of multiple treatment groups, such as surgery, surgery and radiotherapy, surgery and chemotherapy or surgery with chemotherapy and radiotherapy.

## Outcomes and prioritization

The primary outcome will be recurrence-free survival (RFS), local control (LC), failure patterns after treatment, and high-risk factors for recurrence. Patient parameters (such as age), treatment parameters (such as type of surgery, surgical resection margins, and adjuvant treatment), and disease parameters (such as size, histology, and grade) influence treatment. We have multiple parameters to inspect and identify its role in the failure of treatment failure. Failure refers to disease recurrence and can occur locally, regionally or distantly. Identifying the influence of variables on outcomes will help tailor better treatment strategies and reduce future recurrences.

The secondary outcome will be to calculate mean age, geographical distribution, mean tumor size, most common histology, most common grade, survival outcomes such as disease-specific survival (DSS) and overall survival (OS).

## Quality appraisal

The studies selected under the current review be evaluated using quality appraisal tools for quantitative studies produced by the Strengthening the Reporting of Observational Studies in Epidemiology (STROBE) checklist (supplementary file 4). The following topics are appraised: population, method of selection, outcomes, analyses and summary. A copy of the completed checklists will be published with the review results as an additional file.

For case reports and case series, the Joanna Briggs Institute (JBI) Critical Appraisal Checklist for Case Reports (supplementary file 5 and 6) will be used to assess the risk of bias.

## Data Synthesis

Data from all the studies to be included will be extracted by two independent reviewers (AG and RK) using Covidence. A list of biases will be generated, which will be critically evaluated by separate investigators. Discrepancies will be resolved through mutual discussions. Methodological heterogeneity will be evaluated separately by investigators by critically examining the study design. Statistical heterogeneity will be reported using *the I^2^* and *χ^2^ values*. A value of *I^2^* > 60% and *χ^2^* with p < 0.05, was used to assess heterogeneity. The level of interventions and outcome measures will be tabulated to assess the applicability of the meta-analysis.

Categorical variables, such as type of surgery and tumors in each study, will be presented using frequency and percentages. The continuous outcome variables such as resection margins and age will be reported as mean and standard deviation (SD or median with interquartile range (IQR) for continuous variables, depending on the reporting by different studies. The odds ratios (OR) comparing surgical excision without neo/and or adjuvant therapy against surgical excision with neo/and or adjuvant therapy will be reported using 95% confidence interval (CI) and p-value <0.05. Local recurrence and regional and distant metastasis will be plotted using survival curves and hazard ratios (HR) with 95% CI will be calculated for the same. Forest plots for the primary and secondary outcomes will be reported. Further, other clinical variables of interest with adequate data will be reported.

We are interested in determining the natural course of para testicular sarcoma and the possible scope of different interventions will be determined to bring changes in the outcome.

1. Summarizing characteristics of the study
2. Identification of similar vs dissimilar studies
3. Synthesis as per data availability in each study
4. Rules for change in comparator if needed
5. Synthesis of characteristics of studies.

## Ethics and Dissemination

This systematic review does not require ethics approval because published studies with non-identifiable data will be used. No data can be linked to an individual. This protocol complied with the PRISMA-P guidelines. In addition, the findings of the systematic review will be reported according to the PRISMA statement, and will have important implications for epidemiological modelling and research.

## Meta-bias

We will evaluate for outcome reporting bias i.e., whether selective reporting of outcomes is present or not. We will compare the fixed effect estimate against the random effects model to assess the possible presence of small sample bias in the published literature (i.e., in which the intervention effect is more beneficial in smaller studies). In the presence of a small sample bias, the random effects estimate of the intervention is more beneficial than the fixed effect estimate. The potential for reporting bias will be further explored using funnel plots if more than 10 studies are available.

## Limitation

The classification of soft tissue tumors especially sarcomas has undergone major changes in the recent years, mainly due to new knowledge of novel immunohistochemical markers and the identification of specific genetic alterations. Histopathological diagnosis during the publication of various studies may be in disagreement with the present classification, which is a major limitation. Another major limitation is the lack of robust trials and studies with a high level of evidence. Most of the studies are presented in the form of case studies and case series. Case studies have limitations of its own [23], mainly being the publication bias, which means publication of only positive case reports, over-interpretation or misinterpretation of individual case studies.

## Conclusion

Para testicular sarcoma represents a rare and heterogeneous group of lesions where no consensus on the type of surgery and adjuvant treatment is yet established. This systematic review aims to evaluate the patterns of failure and risk factors for recurrence after surgical excision and adjuvant treatment in adult patients with non-metastatic paratesticular sarcoma in the past five decades.

## Additional Files

The additional files for this article can be found as follows:

10.29337/ijsp.145.s1 Supplementary file 1. Keywords and filter for study search.

10.29337/ijsp.145.s2 Supplementary file 2. PRISMA 2009 Flow Diagram.

10.29337/ijsp.145.s3 Supplementary file 3. Data Extraction Form.

10.29337/ijsp.145.s4 Supplementary file 4. STROBE Statement.

10.29337/ijsp.145.s5 Supplementary file 5. JBI Critical Appraisal Checklist for Case Series.

10.29337/ijsp.145.s6 Supplementary file 6. JBI Critical Appraisal Checklist for Case Reports.
