# Supplementary material for: Identifying Patterns of Failure and Risk Factors for Recurrence in Patients of Paratesticular Sarcomas: Protocol of a Systematic Review and Meta-Analysis
Source: Int J Surg Protoc. 2021 May 28;25(1):84–91. doi: 10.29337/ijsp.145 (PMC8162288; doi:10.29337/ijsp.145)
Supplement: Supplementary file 1. — Keywords and filter for study search. [file ijsp-25-1-145-s1.pdf]

Keywords and filter for study search

Pub med

((((((((Paratesticular sarcoma[Title]) OR (spermatic cord sarcoma[Title]))]])) OR (scrotal sarcoma[Title])) NOT (renal[Title])) NOT (retroperitoneal[Title])) NOT (penile[Title])) NOT (vulvar[Title])) NOT (uretheral[Title])) NOT (pediatric[Title])

Web of Science

((((((((Paratesticular sarcoma[Title]) OR (spermatic cord sarcoma[Title])) OR (seminal vesicle sarcoma[Title])) OR (scrotal sarcoma[Title])) NOT (renal[Title])) NOT (retroperitoneal[Title])) NOT (penile[Title])) NOT (vulvar[Title])) NOT (uretheral[Title])) NOT (pediatric[Title])

Scopus

You searched for: TOPIC: (Paratesticular sarcoma) OR TOPIC: (spermatic cord sarcoma) OR TOPIC: (seminal vesicle sarcoma) OR TOPIC: (scrotal sarcoma) NOT TITLE: (renal) NOT TITLE: (retroperitoneal) NOT TITLE: (penile) NOT TITLE: (vulvar) NOT TITLE: (pediatric)

Timespan: 1970-2020. Indexes: SCI-EXPANDED.

Embase

(TITLE-ABS-KEY (paratesticular AND sarcoma) OR TITLE-ABS-KEY (spermatic AND cord AND sarcoma) OR TITLE-ABS-KEY (seminal AND vesicle AND sarcoma) OR TITLE-ABS-KEY (scrotal AND sarcoma) AND NOT TITLE (renal) AND NOT TITLE (retroperitoneal) AND NOT TITLE (penile) AND NOT TITLE (vulvar) AND NOT TITLE (pediatric))

('paratesticular sarcoma':ti,ab,kw OR 'spermatic cord sarcoma':ti,ab,kw OR 'seminal vesicle sarcoma':ti,ab,kw OR 'scrotal sarcoma':ti,ab,kw) NOT renal:ti NOT retroperitoneal:ti NOT penile:ti NOT vulvar:ti NOT paediatric:ti

APPENDIX IV

Standardized Data Extraction Form

Study parameters-

Country—which Country this study was performed.

Country

1. USA USA
2. France
3. Germany
4. U.K U.K
5. Spain Spain
6. Japan Japan
7. India India
8. Italy Italy
9. Morocco
10. Brazil
11. Turkey
12. Other Other

Language- Which language the original study is.

Language

1. English
2. French
3. Japanese
4. Italian
5. Spanish
6. Other Other

Year of publication- when was the study published?

Year of Publication

1. 1970-1980
2. 1981-1990

3. ○ ○ 1991-2000
4. ○ ○ 2001-2010
5. ○ ○ 2011-2020

Age of patient-

Age

1. ○ ○ >18-30
2. ○ ○ >31-40
3. ○ ○ >41-50
4. ○ ○ >51-60
5. ○ ○ >61-70

Disease parameters-

Site- Which subsite of paratestis tumor is arising

1. ○ ○ Spermatic cord
2. ○ ○ Vas deferens
3. ○ ○ Testicular tunics
4. ○ ○ Epididymis
5. ○ ○ Efferent ductules
6. ○ ○ Rete testis

Tumour size: If the size of the tumour is less than 5 cm, 5-10 cm, 10-15 cm or more than 15 cm

Size of Tumor

1. ○ <5cm ○ <5cm
2. ○ ○ 5-10 cm
3. ○ ○ >10-15 cm
4. ○ ○ >15 cm

Laterality of Tumor- - Whether arising from right side of scrotum or left

1. ○ Left ○ Left
2. ○ Right ○ Right

Histology

1. ○ ○ Leiomyosarcoma
2. ○ ○ Rhabdomyosarcoma

3. Liposarcoma
4. Ewing Sarcoma
5. De-differentiated Liposarcoma
6. Lipoleiomyosarcoma
7. Malignant Fibrous Histiocytoma
8. Other Other

Grade

1. I I
2. II II
3. III III
4. IV IV

Treatment parameters-

Type of Surgery

1. Radical orchiectomy with high ligation of the spermatic cord
2. Radical orchiectomy Only
3. Hemiscotectomy
4. Radical orchiectomy with RPLND
5. Radical orchiectomy with Inguinal LND
6. Radical orchiectomy with RPLND and Inguinal LND
7. Other Other

Resection Margins- Of surgery

1. R0 R0
2. R1 R1
3. R2 R2

Adjuvant treatment- if adjuvant treatment given whether it is radiotherapy, chemotherapy or both.

1. Chemotherapy
2. Radiotherapy
3. Both Both
4. None None

Follow up period

1. <6 m <6 m
2. 6-12 m
3. >12-24 m

4. >24-36 m
5. >36-48 m
6. >48-60 m
7. >60 m >60 m

Recurrence Site

1. Local Local
2. Regional
3. Distant
4. None None

Last status

1. Healthy
2. Recurrence
3. Dead Dead

APPENDIX V

STROBE Statement—Checklist of items that should be included in reports of ***cross-sectional studies***

	Item No	Recommendation
**Title and abstract**	1	(*a*) Indicate the study's design with a commonly used term in the title or the abstract
		(*b*) Provide in the abstract an informative and balanced summary of what was done and what was found

**Introduction**		
Background/rationale	2	Explain the scientific background and rationale for the investigation being reported
Objectives	3	State specific objectives, including any prespecified hypotheses
**Methods**		
Study design	4	Present key elements of study design early in the paper
Setting	5	Describe the setting, locations, and relevant dates, including periods of recruitment, exposure, follow-up, and data collection
Participants	6	(*a*) Give the eligibility criteria, and the sources and methods of selection of participants
Variables	7	Clearly define all outcomes, exposures, predictors, potential confounders, and effect modifiers. Give diagnostic criteria, if applicable
Data sources/ measurement	8*	For each variable of interest, give sources of data and details of methods of assessment (measurement). Describe comparability of assessment methods if there is more than one group
Bias	9	Describe any efforts to address potential sources of bias
Study size	10	Explain how the study size was arrived at
Quantitative variables	11	Explain how quantitative variables were handled in the analyses. If applicable, describe which groupings were chosen and why
Statistical methods	12	(*a*) Describe all statistical methods, including those used to control for confounding
		(*b*) Describe any methods used to examine subgroups and interactions
		(*c*) Explain how missing data were addressed
		(*d*) If applicable, describe analytical methods taking account of sampling strategy
		(*e*) Describe any sensitivity analyses
**Results**		
Participants	13*	(a) Report numbers of individuals at each stage of study—eg numbers potentially eligible, examined for eligibility, confirmed eligible, included in the study, completing follow-up, and analysed
		(b) Give reasons for non-participation at each stage
		(c) Consider use of a flow diagram
Descriptive data	14*	(a) Give characteristics of study participants (eg demographic, clinical, social) and information on exposures and potential confounders
		(b) Indicate number of participants with missing data for each variable of interest
Outcome data	15*	Report numbers of outcome events or summary measures

Main results	16	(*a*) Give unadjusted estimates and, if applicable, confounder-adjusted estimates and their precision (eg, 95% confidence interval). Make clear which confounders were adjusted for and why they were included
		(*b*) Report category boundaries when continuous variables were categorized
		(*c*) If relevant, consider translating estimates of relative risk into absolute risk for a meaningful time period
Other analyses	17	Report other analyses done—eg analyses of subgroups and interactions, and sensitivity analyses
**Discussion**		
Key results	18	Summarise key results with reference to study objectives
Limitations	19	Discuss limitations of the study, taking into account sources of potential bias or imprecision. Discuss both direction and magnitude of any potential bias
Interpretation	20	Give a cautious overall interpretation of results considering objectives, limitations, multiplicity of analyses, results from similar studies, and other relevant evidence
Generalisability	21	Discuss the generalisability (external validity) of the study results
**Other information**		
Funding	22	Give the source of funding and the role of the funders for the present study and, if applicable, for the original study on which the present article is based

*Give information separately for exposed and unexposed groups.

APPENDIX VI

**P. The Joanna Briggs Institute (JBI) Critical Appraisal Checklist for Case Reports (last amended in 2017)**				
Major Components	Response options			
1. Were patient’s demographic characteristics clearly described?	Yes	No	Unclear	Not applicable

2. Was the patient's history clearly described and presented as a timeline?	Yes	No	Unclear	Not applicable
3. Was the current clinical condition of the patient on presentation clearly described?	Yes	No	Unclear	Not applicable
4. Were diagnostic tests or assessment methods and the results clearly described?	Yes	No	Unclear	Not applicable
5. Was the intervention(s) or treatment procedure(s) clearly described?	Yes	No	Unclear	Not applicable
6. Was the post-intervention clinical condition clearly described?	Yes	No	Unclear	Not applicable
7. Were adverse events (harms) or unanticipated events identified and described?	Yes	No	Unclear	Not applicable
8. Does the case report provide takeaway lessons?	Yes	No	Unclear	Not applicable
Overall appraisal: Include ☐ Exclude ☐ Seek further info ☐				
