# Supplementary material for: Identifying Patterns of Failure and Risk Factors for Recurrence in Patients of Paratesticular Sarcomas: Protocol of a Systematic Review and Meta-Analysis
Source: Int J Surg Protoc. 2021 May 28;25(1):84–91. doi: 10.29337/ijsp.145 (PMC8162288; doi:10.29337/ijsp.145)
Supplement: Supplementary file 3. — Data Extraction Form. [file ijsp-25-1-145-s3.pdf]

Data Extraction Form

Study parameters

Country this study was performed

1. ○ USA
2. ○ France
3. ○ Germany
4. ○ U.K
5. ○ Spain
6. ○ Japan
7. ○ India
8. ○ Italy
9. ○ Morocco
10. ○ Brazil
11. ○ Turkey
12. ○ Other

Which language the original study is

1. ○ English
2. ○ French
3. ○ Japanese
4. ○ Italian
5. ○ Spanish
6. ○ Other

When was the study published

1. ○ 1970-1980
2. ○ 1981-1990
3. ○ 1991-2000
4. ○ 2001-2010
5. ○ 2011-2020

Year (in number)

Age

1. ≥18-20
2. 21-30
3. 31-40
4. 41-50
5. 51-60
6. 61-75

Age (in number)

Disease parameters

Which subsite of paratestis tumor is arising?

1. Spermatic cord
2. Vas deferens
3. Testicular tunics
4. Epididymis
5. Efferent ductules
6. Rete testis
7. None of the above

Laterality of tumor

1. Left
2. Right

Size of tumor

1. <5cm
2. 5-10 cm
3. >10-15 cm
4. >15 cm
5. Not mentioned

Size of tumor (in cm)

Histology

1. Leiomyosarcoma
2. Rhabdomyosarcoma
3. Liposarcoma
4. Ewing Sarcoma
5. De-differentiated Liposarcoma
6. Lipoleiomyosarcoma
7. Malignant Fibrous Histiocytoma
8. Other

FNLCC Grade

1. I
2. II
3. III
4. Not mentioned

Two grade system

1. Low Grade
2. High Grade
3. Not mentioned

Treatment parameters

Type of Surgery

1. Radical orchiectomy with high ligation of the spermatic cord
2. Radical orchiectomy only
3. Hemiscrotectomy
4. Radical orchiectomy with RPLND
5. Radical orchiectomy with inguinal LND
6. Radical orchiectomy with RPLND and inguinal LND
7. Other

Resection Margins

1. ◯ R0
2. ◯ R1
3. ◯ R2
4. ◯ Not mentioned

Adjuvant treatment

Adjuvant treatment

1. ◯ Chemotherapy
2. ◯ Radiotherapy
3. ◯ Both
4. ◯ None
5. ◯ Not mentioned

Dose

Follow up period

1. ◯ <6 m
2. ◯ 6-12 m
3. ◯ >12-24 m
4. ◯ >24-36 m
5. ◯ >36-48 m
6. ◯ >48-60 m
7. ◯ >60 m

Follow up period (in months)

Recurrence Site

1. ◯ Local
2. ◯ Regional

3. ◯ Distant
4. ◯ None

Last status

1. ◯ Healthy
2. ◯ Recurrence
3. ◯ Dead
